# New music combination promotes neuroimmune homeostasis and stress relief

**DOI:** 10.3389/fnhum.2026.1730014

**Published:** 2026-02-17

**Authors:** Yinji Liu, Zhenyu Han, Li Huang, Ying Chen, Ziqi Men, Wenhao Xu, Youpeng Sun, Feixiang Li, Pinhong Chen, Zhiding Wang, Lubin Wang, Gencheng Han

**Affiliations:** 1 Beijing Institute of Basic Medical Sciences, Beijing, China; 2 Fourth Military Medical University, Xi’an, Shaanxi, China; 3 Emei Rehabilitation and Recuperation Center, Leshan, China

**Keywords:** cortisol, heart rate variability, IgA, music, neuroimmune homeostasis

## Abstract

**Introduction:**

Music has been widely used for disease intervention, while the underlying mechanisms remain to be determined. This study explored whether our novel music combination, which is composed of four pieces of music, can promote neuroimmune homeostasis and then relieve stress.

**Methods:**

Fifty-five participants were enrolled in the study, and they underwent three separate music therapy sessions or periods of rest. Saliva and blood samples were collected, cognitive task testing was conducted, and electrocardiographic (ECG) data were recorded.

**Results:**

The results showed that our new music combination increased the heart rate variability (HRV) index [RMSSD, PNN20, PNN50, and high-frequency (HF)/NU] while decreasing LF/NU, which suggested restoration of balance between the sympathetic and parasympathetic activity and relieved stress. In addition, participants in the music group had lower Balloon Analogue Risk Task (BART) test results and higher Multiple Object Tracking (MOT) task test results, suggesting increased attention and stress relief. Music therapy also increased the IgA while decreasing cortisol concentrations.

**Discussion:**

This study reveals that our novel music combination may relieve stress by promoting neuroimmune homeostasis, which sheds new light on the mechanisms of music therapy and suggests new approaches for intervention.

## Highlights

- New music combination increases heart rate variability and reduces stress.
- New music combination promotes neuroimmune homeostasis.
- New music combination boosts attention, as shown in BART and MOT.

## 1 Introduction

Music therapy is a therapeutic modality that promotes both physical and mental health (Bruscia, 2018). With its merits of low cost, no side effects, and low restrictions, there has been a growing interest in the use of music in healthcare. Studies have shown that music interventions can exert a positive influence in multiple scenarios (Ting et al., 2023), such as reducing the level of depression (Aalbers et al., 2017), reducing anxiety (Dallı et al., 2023), improving the cognitive function of dementia patients (Pauwels et al., 2014), relieving the pain during surgery (Dallı et al., 2023; Lee, 2016), exerting a beneficial impact on the sleep quality and pain of patients with coronary heart disease (Bradt et al., 2013), and reducing the blood pressure of patients with chronic hypertension (Bekiroğlu et al., 2013), and assisting in the treatment of infectious diseases such as COVID-19 (Mol et al., 2021). Although some reports showed that music may promote health by regulating neuroimmune response (Chanda and Levitin, 2013; Zhang et al., 2021), some direct evidence is still needed.

Different types of music have been used for music therapy. Western classical music, such as Mozart’s K.448 Piano Sonata (Di Cesare et al., 2022; Wang et al., 2025), and Chinese classical music (Li et al., 2010, 2022), such as Five Elements music (Tao et al., 2016), all exhibit different characteristics. Therefore, it is intriguing to explore the effects of combined music that incorporates both Western and Chinese traditional elements.

Reports showed that music can affect central nervous system function, but whether it works by affecting the peripheral nervous system still needs to be proven. The peripheral autonomic nervous system is divided into sympathetic and parasympathetic nerve branches, which dynamically balance each other under normal conditions. When exposed to a stressor, the sympathetic nervous system becomes more active than the parasympathetic nerve, causing changes in some physiological indicators. Conversely, when living in a relaxed state, the parasympathetic nervous system takes over (Benarroch, 2020). Recently, vagus nerve (a branch of the parasympathetic nerve) stimulation has been widely used in the treatment of clinical diseases. The cholinergic anti-inflammatory pathway (CAP) has been used to explain the mechanisms of vagus nerve stimulation. Although it is known that music can make people calm down, how music relieves stress via parasympathetic nerve stimulation, i.e., via balancing the sympathetic and parasympathetic branches of the autonomic nerve system, is an area of great interest for further research (Baccarani et al., 2023).

Communication between the nervous and the immune system is crucial for controlling inflammation (Dedoncker et al., 2021). Besides the cholinergic anti-inflammatory pathway, the autonomic nervous system may also regulate inflammation by activating the hypothalamic-pituitary-adrenal axis. The autonomic nervous system notifies the brain of peripheral inflammation and then reflexively suppresses this inflammation by producing cortisol (Johnston and Webster, 2009; Pavlov and Tracey, 2012). In such a scenario, whether music regulates endocrine-immune response via the autonomic nervous system is an important question waiting to be explored.

Autonomic nervous system activity can be measured by heart rate variability (HRV). HRV is a non-invasive electrocardiographic (ECG) index of the autonomic control of the heart, which refers to the heartbeat interval changes, including time domain (RMSSD, PNN20, and PNN50) (Kim et al., 2018), frequency domain [high-frequency (HF), LF, and VLF band] (Arakaki et al., 2023), and non-linear index measuring [standard deviation 1 (SD1)] (Shaffer and Ginsberg, 2017). It reflects the efferent autonomic nervous system activity of the heart and is a quantitative indicator of autonomic regulation. Studies have found a significant correlation between actual parasympathetic nerve activity and HRV, in which parasympathetic nerve activity can be indexed by measuring a patient’s HRV (Kim et al., 2018). The application of HRV to evaluate the autonomic nervous system in music therapy is worth exploring.

Balloon Analogue Risk Task (BART) is a test used to evaluate stressful conditions via decision-making. In the BART, participants inflate computer-simulated balloons and earn a certain amount of money through each pump (Lejuez et al., 2003). Meanwhile, each pump increases the risk of balloon explosion. If the balloon explodes, all the money earned so far will be lost. Because decisions in daily life involve both rewards and loss possibilities, this task is an ecologically effective paradigm for simulating adventurous behavior under experimental conditions (Hengen and Alpers, 2021). The multiple-object tracking (MOT) task involves a fixed number of identical elements (such as circular dots) displayed on a computer screen. Elements can be targets or interfering factors. At first, all elements are stationary, indicating the number of target objects. All elements then undergo random motion, and participants need to maintain their attention to the prompt object. At the end of the experiment, all elements stop moving, and participants need to indicate the target element (Alnawmasi and Khuu, 2022). The MOT task can serve as an effective tool for assessing an individual’s level of attention. BART and MOT tasks are two classical tests evaluating stress or attention.

This study aimed to assess the effects of our new music combination on stress relief in individuals engaged in high-intensity work. We found that our new music combination can relieve stress, indicated by increased activation of the parasympathetic nerve (increased HRV), lower BART, and higher MOT task performance. Additionally, the new music combination promotes homeostasis of the endocrine-immune axis through increasing IgA levels while decreasing cortisol production. Music intervention may function as a stress reliever to improve health.

## 2 Materials and methods

### 2.1 Participants

Fifty-five participants (all males) volunteered for the study. All participants worked in confined environments that required prolonged concentration. Based on their medical history assessments, all study participants are free of cardiovascular disease, hearing impairment, neurological disorders, and other diagnosed diseases. None of the volunteers is taking corticosteroids or other hormone medications. Participants were all males to control for the effects of the menstrual cycle on hormonal and autonomic function. Participants were randomly assigned to the control (rest) group and the experimental (music) group, of which 31 were in the experimental group, with a mean age of 29.8 ± 5.1 years. A total of 24 participants were in the control group, with a mean age of 28.8 ± 4.7 years.

This study was approved by the Ethics Committee of the Beijing Institute of Basic Medical Sciences, and all methods were performed following the relevant guidelines and regulations. Participants received saliva and blood sampling materials, along with oral and written instructions. Before data collection, the purpose of the study was thoroughly explained to each volunteer following the Declaration of Helsinki, and all participants signed informed consent after understanding the content of the study.

### 2.2 Music

**FIGURE 1:**
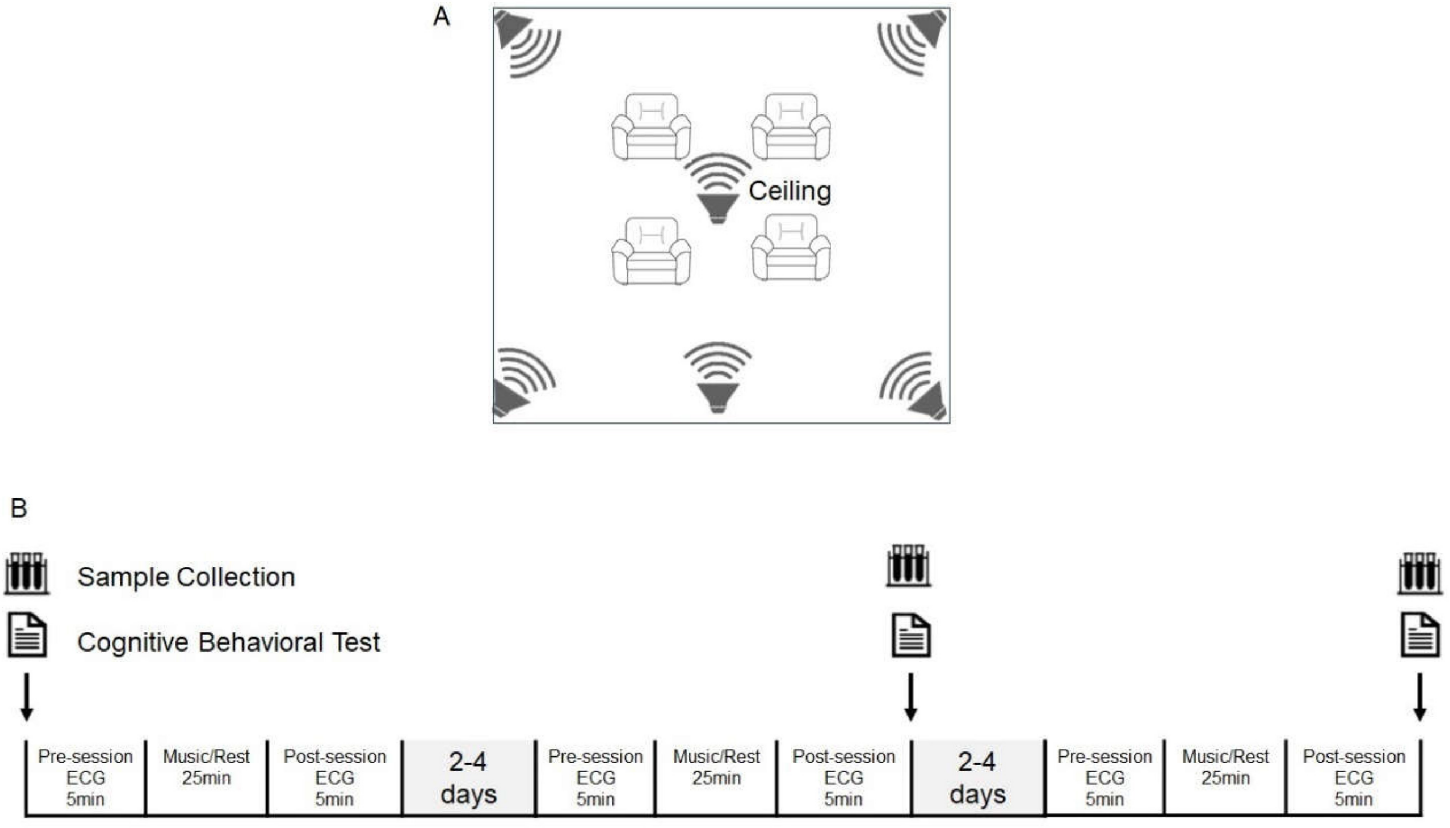
Schematic diagram of the experimental scenario and procedure. **(A)** The experimental scenario. **(B)** The experimental procedure.

For the intervention music, we have combined four musical pieces and arranged them based on the logic of emotional progression in the following sequence: (1) Monday, a piano composition by the Italian pianist Ludovico Einaudi, simple tonal harmony, limited chord progressions with a duration of 5 min and 57 s. (2) ChunJiangHuaYueYe, a renowned Chinese classical music piece, pentatonic scale core, modal harmony with subtle ornamentation. The version selected for this experiment was performed by the Central Ethnic Orchestra, having a duration of 9 min and 19 s. (3) Sonata for 2 Pianos in D Major (K.448), a piano sonata by Mozart, the European classical music composer, western tonal harmony, heptatonic scale (7-note scale) with a duration of 9 min and 23 s. (4) JiangNanHao, a Chinese folk music ensemble that exhibits typical characteristics of Chinese Jiangnan music, pentatonic scale core, modal harmony with subtle ornamentation. The version used is performed by the ethnic band Jiangsu Song and Dance Troupe, with a duration of 4 min and 50 s. The music was sourced from high-quality online recordings. It was played using a six-channel surround sound system in the therapy room (Figure 1A), and the volume was maintained at 50–70 dB (as measured by a Decibel meter).

### 2.3 Design and procedure

Participants were required to participate in three sessions, each separated by an interval of 2–4 days (Figure 1B). All experiments were conducted every afternoon.

First session: (1) Collection of Biological Samples: Biological samples were collected from the volunteers, including 5 ml of blood and 5 ml of saliva. Cognitive Behavioral Tests: Participants underwent cognitive behavioral tests, which comprised the Balloon Analog Risk Task, the Multiple Object Tracking Task, and working memory tasks. (2) Pre-session ECG Data Collection: ECG data were recorded from the participants for a 5-min resting state prior to the session. (3) Experimental Procedures: Music group: The volunteers in the music group listened to four music tracks while their ECG was being recorded. They were instructed to relax and rest during this period. Sleeping, conversation, and the use of mobile phones were prohibited. Control Group: The volunteers in the control group sat still for 25 min while their ECG was being recorded. They were also instructed to relax and rest, with the same prohibitions applying. (4) Post-session ECG Data Collection: Following the session, ECG data were once again collected from the participants for a 5-min resting state.

Second and third sessions: The contents of the second and third sessions were the same as the content of the first session, but the sequence of steps was altered. Specifically, the steps were changed to follow the sequence 2-3-4-1 of the first session, meaning that ECG data were measured first, followed by biological sample collection and cognitive task testing (Figure 1B).

#### 2.3.1 Biological sample collection and assay

During each session, blood and saliva samples were collected from each volunteer. To guarantee the accuracy of the sessions, volunteers were instructed to ensure sufficient sleep throughout the experimental period. Additionally, they were required to refrain from consuming alcohol, caffeinated food or beverages, and engaging in strenuous activities for 24 h before each session.

For blood samples, a professional nurse conducted the collection, and these samples are then sent to a laboratory for serum isolation. For saliva samples, a volunteer should rinse the oral debris with clean water 30 min before sampling. During this period, it is essential not to eat, chew gum, or drink water. Both saliva samples and serum samples are stored at −80 °C until biochemical analysis is performed.

Tested by LEGENDplex (Lehmann et al., 2019), a technique for bead-assisted multiplex cytokine profiling by flow cytometry to detect cortisol and IgA in serum and saliva samples of volunteers. Exported data files generated were analyzed using the LEGENDplex Data Analysis Software (BioLegend).

#### 2.3.2 Cognitive task

##### 2.3.2.1 Balloon Analogue Risk Task (BART)

In this task, the volunteers perform the operation by tapping a balloon located in the center of the screen. With each tap, the balloon inflates, and the risk of it bursting increases correspondingly, while the reward also rises in accordance. However, if the balloon bursts during the inflating process, the volunteer will forfeit a portion of the accumulated reward. During the execution of the task, volunteers constantly assess the possible reward of inflating the balloon versus the potential penalty of it bursting. Therefore, an individual’s propensity for risk can be gauged by observing whether they inflate the balloon and how frequently they do so.

##### 2.3.2.2 Multiple Object Tracking (MOT)

The Multiple Object Tracking (MOT) task (Pylyshyn and Storm, 1988) consists of three stages: cue, tracking, and reaction. In the cue stage, multiple green particles are displayed on the screen, with some of them being marked as target particles in red. During the tracking phase, the color of the target particles changes to green, and all particles began to move randomly and independently, requiring the volunteer to track the targets that were marked during the cue stage. In the reaction phase, after tracking for a specified period, the motion ceases, and the volunteers are prompted to select which objects are the target particles.

#### 2.3.3 ECG

Electrocardiographic is collected using a dynamic electrocardiograph. The group multi-channel exercise ECG monitoring system (BW-ECG-CHA, Shanxi Yikang Xinyue Medical Equipment Co., LTD) was used to monitor the volunteers in both the music group and the control group in real-time, which sampling rate is 128 Hz. This system recorded the volunteers’ data and analyzed the HRV data, which included time-domain measurements, frequency-domain measurements, and non-linear measurements.

### 2.4 Statistical analysis

Statistical analyses were performed using GraphPad Prism 9.5 software. Technical statistics, as a method of statistical analysis, were conducted using paired-test and unpaired-test to analyze and verify the effects of music therapy during follow-up. Data are expressed as average ± standard deviation. Statistical significance was set at *p* < 0.05.

## 3 Results

In the music group, data from 31 participants were collected. Among them, 31 participants completed the 1st session, 23 participants completed the 2nd session, and 22 participants completed the 3rd session. In the control group, data from 24 participants were collected. Among them, 24 participants completed the 1st session, 24 participants completed the 2nd session, and 22 participants completed the 3rd session.

### 3.1 Music stress relief effect on volunteers’ HRV

#### 3.1.1 Time-domain measurements

**FIGURE 2:**
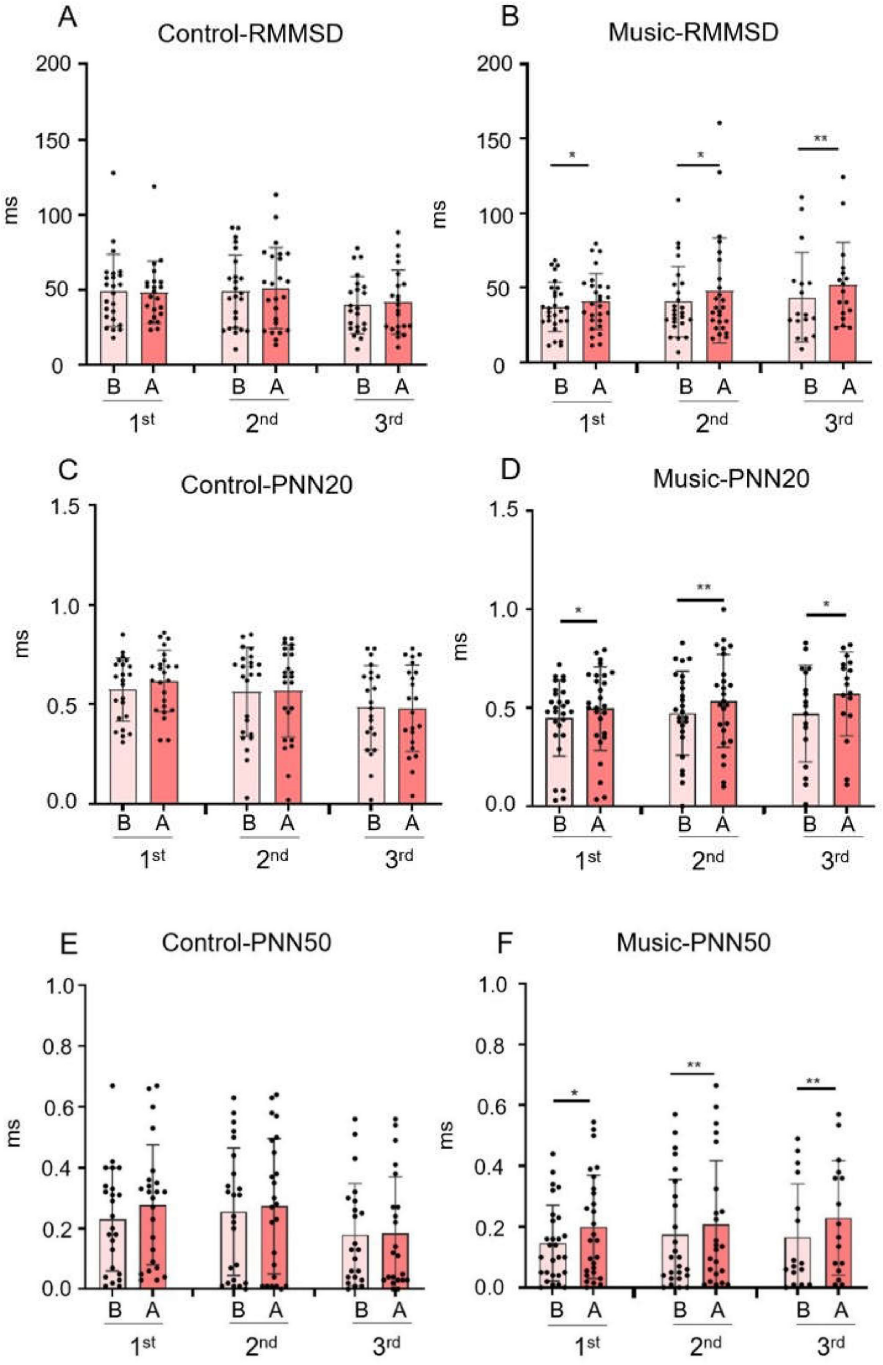
The graph of the statistical results of time-domain measurements of heart rate variability (HRV). (In the graph, B represents the values measured before each intervention in both the control group and music group, while A represents the values measured after each intervention in both groups). **(A)** RMSSD of the control group. **(B)** RMSSD of the music group. **(C)** PNN20 of the control group. **(D)** PNN20 of the music group. **(E)** PNN50 of the control group. **(F)** PNN50 of the music group. (*p < 0.05, ** *p* < 0.01).

The RMSSD reflects the beat-to-beat variance in HR and is the primary time-domain measure used to estimate the parasympathetic-mediated changes reflected in HRV (Supplementary Table 1) (Shaffer et al., 2014). The results showed that RMSSD in the music group (Figure 2B) was higher after each music therapy session than before music therapy (*p* = 0.027; *p* = 0.0244; *p* = 0.0045). The control group (Figure 2A) showed no significant difference in RMSSD changes before and after the three sessions.

PNN20 in the music group was higher after each music therapy session than before music therapy (Figure 2D; *p* = 0.0382; *p* = 0.0012; *p* = 0.0105). However, the proportion of PNN20 in the control group (Figure 2C) did not change significantly after each session.

PNN50 showed no significant change before and after the sessions in the control group (Figure 2E), whereas in the music group (Figure 2F), PNN50 increased after each music therapy (*p* = 0.0290; *p* = 0.0059; *p* = 0.0080).

According to the experimental results (Figure 2), compared with the control group, the measured values of RMSSD, PNN20, and PNN50 of volunteers in the music group all showed increases, indicating that they had a superior parasympathetic nervous system, which indicates parasympathetic nerve activation during listening to music.

#### 3.1.2 Frequency-domain measurements

**FIGURE 3:**
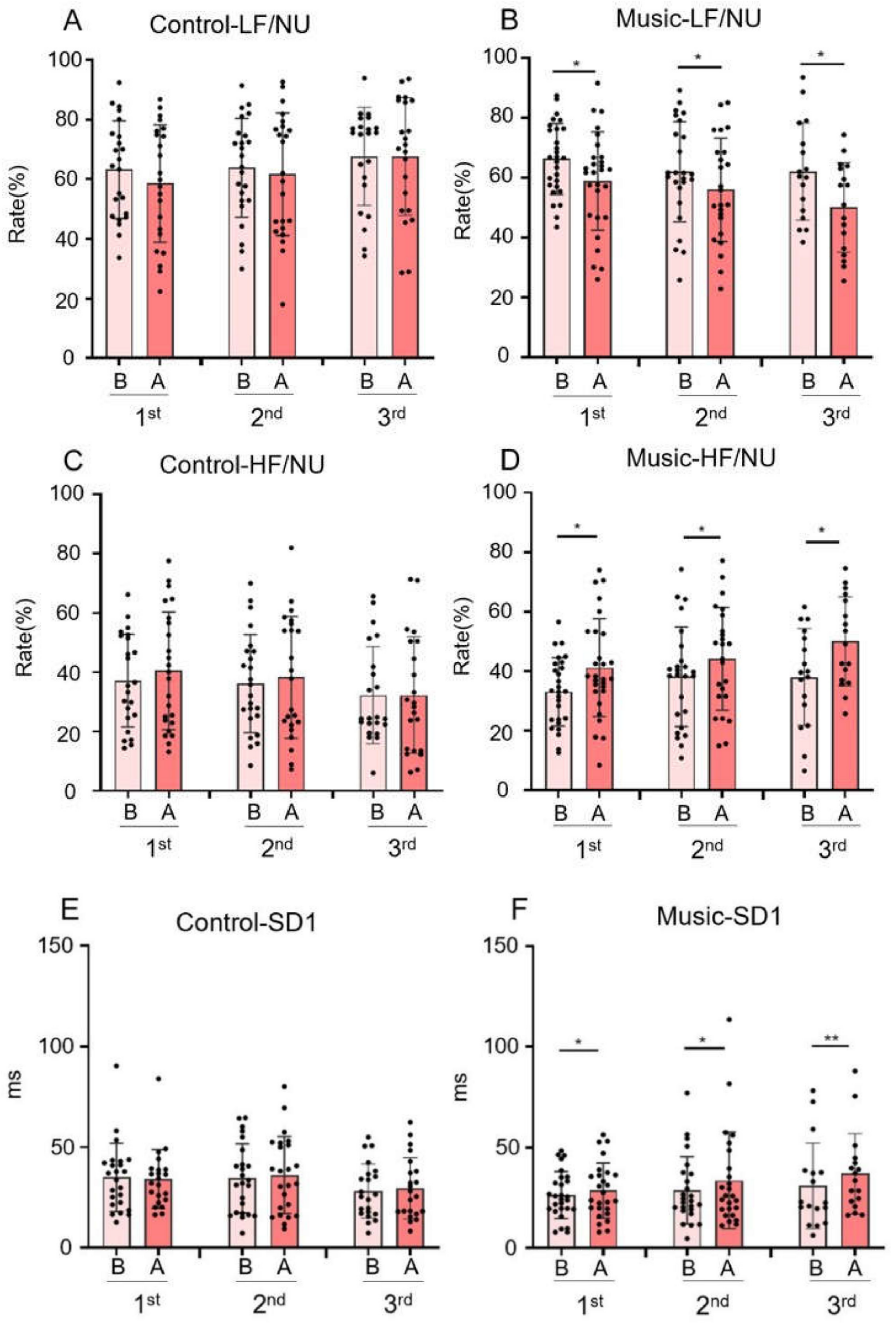
The graph of the statistical results of frequency-domain measurements and non-linear measurements of heart rate variability (HRV). (In the graph, B represents the values measured before each intervention in both the control group and music group, while A represents the values measured after each intervention in both groups). **(A)** LF norm of the control group. **(B)** LF norm of the music group. **(C)** High-frequency (HF) norm of the control group. RMSSD of the music group. **(D)** HF norm of the control group. **(E)** Standard deviation 1 (SD1) of the control group. **(F)** SD1 of the music group. (*p < 0.05, ** *p* < 0.05).

The results LF/NU (LF power in normalized units, calculated as LF/(total power–VLF) × 100) showed that the LF/NU in the music group (Figure 3B) was lower after each music therapy than before music therapy (*p* = 0.0318; *p* = 0.0309; *p* = 0.0101). However, there was no significant difference in LF/NU values in the control group (Figure 3A) before and after three breaks.

As shown in the figure, the HF/NU (HF power in normalized units, calculated as HF/(total power–VLF) × 100) in the control group (Figure 3C) did not change significantly across the three sessions. In contrast, the HF/NU in the music group (Figure 3D) was higher after each music therapy than before (*p* = 0.0147; *p* = 0.0309; *p* = 0.0101).

HF is a measure of parasympathetic nervous system activity, as it reflects parasympathetic nerve activity, while LF reflects sympathetic nervous activity, which dominates autonomic nervous activity. As can be seen from the figure (Figure 3), it is very obvious that the ratio of LF/NU in the music group is decreased; whereas the HF/NU value measured in this group is significantly increased compared with the control group. This indicates that music has the potential to impact the balance of autonomic nervous activity, potentially activating the parasympathetic nervous system and reducing the sympathetic nervous system activity, with the parasympathetic nerve response being dominant.

#### 3.1.3 Non-linear measurements

SD1 in the control group (Figure 3E) did not change significantly across. In contrast, in the music group (Figure 3F), SD1 was significantly higher after each music treatment session compared to before the treatment (*p* = 0.0180; *p* = 0.0227; *p* = 0.0044). The non-linear metric SD1 is equivalent to the RMSSD, which reflects short-term HRV. A higher SD1 value indicates enhanced parasympathetic function during music listening.

### 3.2 Music stress relief effect on volunteers’ cognition tasks

#### 3.2.1 BART

**FIGURE 4:**
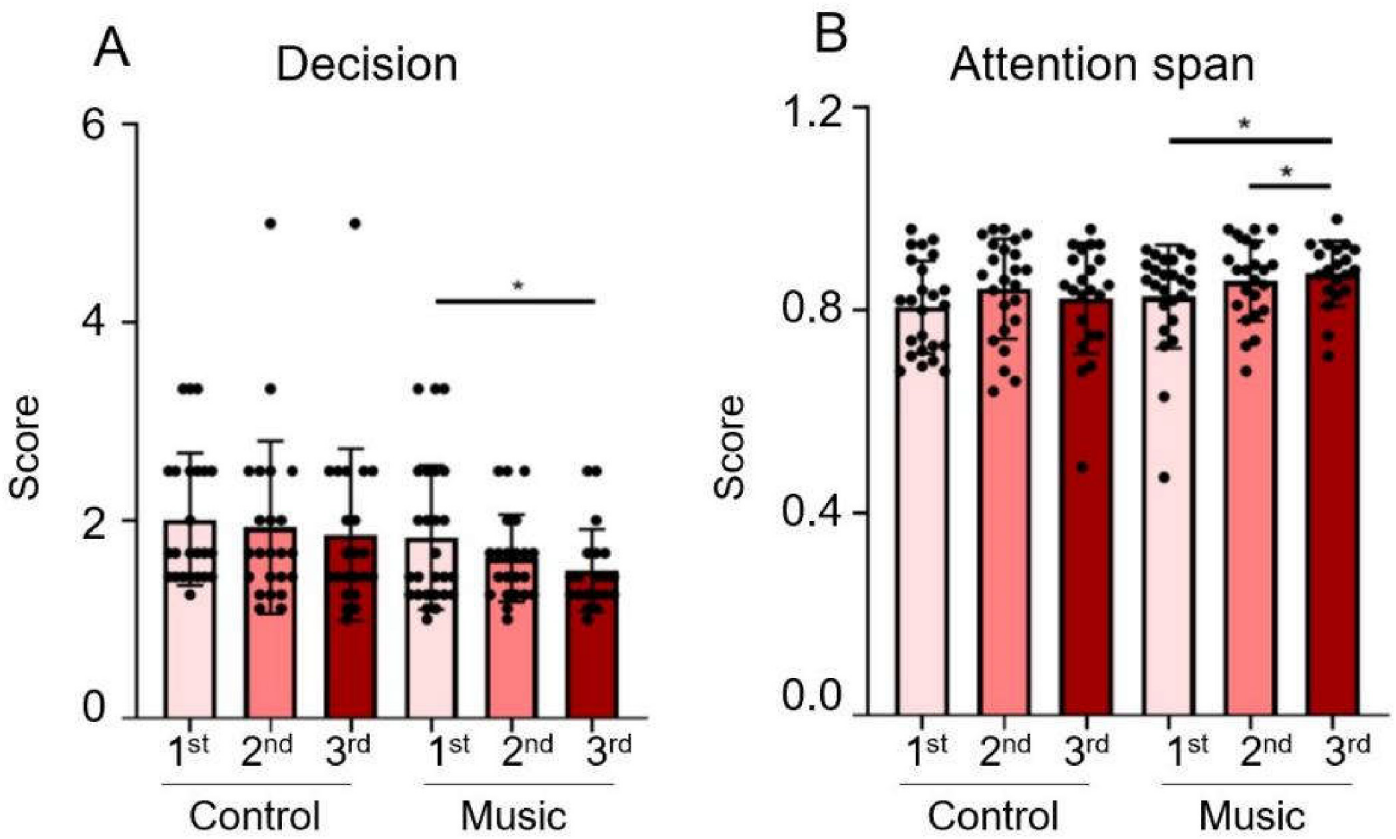
The graph of the statistical results of different tasks. **(A)** Balloon Analogue Risk Task (BART). **(B)** The multiple-object tracking (MOT) task. (*p < 0.05).

The BART (Lejuez et al., 2002) can be utilized as a measurement tool to investigate the relationship between anxiety, stress, and risk-taking behavior. When compared with the control group, the BART scores of the volunteers in the music group (Figure 4A) gradually decreased over the course of three music therapy sessions. Specifically, the measurement results of the volunteers after the final music therapy session were significantly lower than those after the first session, indicating more decisive decision-making (*p* = 0.0491). Conversely, there was no significant change in the control group. Volunteers who experience excessive pressure or have an anxiety tendency are more prone to biases in their perception of risk behavior, leading them to overestimate the actual risk and exhibit higher risk aversion behavior (Kim et al., 2020). The results of the volunteers’ decision scores indicate that music can influence their decision-making and risk-taking behavior.

#### 3.2.2 MOT

This task can reflect the volunteer’s level of attention and attention span to some extent (Drew et al., 2012). According to the MOT task (Figure 4B), the control group showed no significant trend across the three sessions, while the volunteers in the music group gradually improved their attention during the three music therapy sessions, with a significant increase in attention after the third session compared to both the second and the first sessions (*p* = 0.0151; *p* = 0.0216). It is observable that, compared with the control group, volunteers in the music group exhibited improved attention after three interventions.

### 3.3 Music stress relief effect on volunteers’ endocrine-immune axis

#### 3.3.1 IgA

**FIGURE 5:**
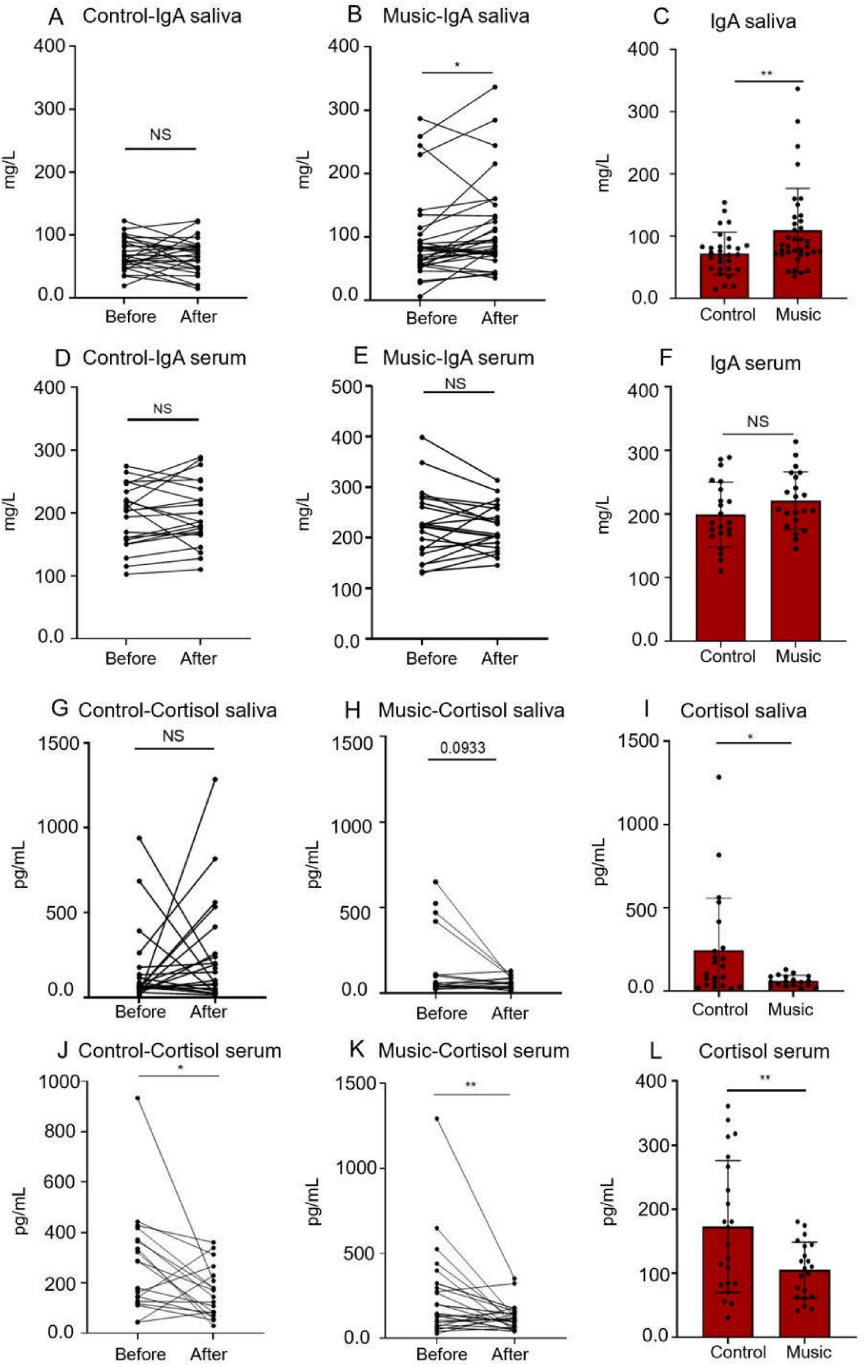
The graph of the statistical results of IgA and cortisol in saliva and serum. **(A)** The results of saliva IgA in the control group before and after the experiment. **(B)** The results of saliva IgA in the music group before and after the experiment. **(C)** After three experiments, the salivary IgA was compared between the music group and the control group. **(D)** The results of serum IgA in the control group before and after the experiment. **(E)** The results of serum IgA in the control group before and after the experiment. **(F)** After three experiments, the serum IgA was compared between the music group and the control group. **(G)** The results of saliva cortisol in the control group before and after the experiment. **(H)** The results of saliva cortisol in the music group before and after the experiment. **(I)** After 3rd session, the salivary cortisol was compared between the music group and the control group. **(J)** The results of serum cortisol in the control group before and after the experiment. **(K)** The results of serum cortisol in the control group before and after the experiment. **(L)** After three experiments, the serum cortisol was compared between the music group and the control group. (*p < 0.05, ** *p* < 0.01).

To evaluate the immune function, the saliva collected before and after rest or music therapy was detected, respectively. As shown in Figure 4, the IgA level in the saliva of the volunteers in the music group (Figure 5B) increased after three experiments (*p* = 0.0224), whereas the IgA level in the saliva of the control group (Figure 5A) did not change significantly. When the results of the two groups were compared (Figure 5C), the IgA levels in the music group were significantly higher than those in the control group (*p* = 0.0093). There was no significant change in serum IgA in either the control group or the music group (Figures 5D,E), and no significant difference was observed between the two groups when compared (Figure 5F). The saliva tests of the volunteers indicated that music can elevate IgA levels, which boosts immune function.

#### 3.3.2 Cortisol

To evaluate the endocrine function, the levels of cortisol in serum and saliva were compared between the control group and the music group before and after the experiment. As shown in Figure 4, the salivary cortisol levels in the control group (Figure 5G) remained relatively stable, whereas the salivary cortisol levels in the music group (Figure 5H) demonstrated a decreasing trend, although this difference was not statistically significant. However, upon comparing the salivary cortisol levels of the two groups after the experiment (Figure 5I), it was found that the music group had significantly lower levels than the control group (*p* = 0.0184). The levels of cortisol in the serum of the volunteers decreased in both the control and music groups (Figures 5J,K). Specifically, when compared with the control group (Figure 5L), the serum cortisol levels in the music group were significantly lower (*p* = 0.0078). Both blood serum and saliva measurements suggest that music could have a beneficial effect by lowering cortisol levels.

## 4 Discussion

Studies have found a significant correlation between actual parasympathetic nerve activity and HRV (Kim et al., 2018). Here, our data showed that HRV can be used as a valuable indicator of how music affects neural function. The time-domain, frequency-domain, and non-linear measurements of HRV are standard clinical parameters (1996). Following a music intervention, higher RMSSD, PNN20, PNN50, HF/NU, and SD1 values, along with lower LF/NU values, indeed reflect the balance of the autonomic nervous system, specifically activating the parasympathetic nervous system and giving it a predominant role. It is well-known that listening to music can relieve stress and anxiety by regulating the nervous system. Here, we provided new direct evidence that the parasympathetic nervous system can be activated, as evidenced by increased HRV (Bravo et al., 2011; Richer et al., 2022).

The activation of the parasympathetic nervous system not only reflects the reversal of stress states, but in recent years, the anti-inflammatory properties of this nerve branch have also garnered significant attention (Bonaz et al., 2021). Brain and visceral interactions within the autonomic nervous system are crucial. Specifically, the parasympathetic nervous system, which contains approximately 80% of afferent fibers and 20% of efferent fibers, plays a variety of key roles in the homeostatic regulation of visceral function. Recent data suggest that the parasympathetic nervous system exhibits anti-inflammatory effects and is a major component of the neuroendocrine-immune axis (Guyot et al., 2019; Johnston and Webster, 2009). This parasympathetic function is mediated through several pathways, some of which remain controversial. One such pathway is the anti-inflammatory hypothalamic-pituitary-adrenal (HPA) axis, which stimulates the adrenal glands to release cortisol (Lightman et al., 2020). There is a close relationship between the HPA axis and the autonomic nervous system. Our experimental results also observed that cortisol levels in the saliva and serum of volunteers in the music group were lower than those in the control group after three music interventions (Figure 5), indicating that music may alleviate inflammatory stimuli in the body, although the specific mechanism needs further investigation.

The second pathway, known as the cholinergic anti-inflammatory pathway, originates in the abdominal ganglion, down from the vagus nerve to the sympathetic nerve (Borovikova et al., 2000), and reaches the spleen (Zhang et al., 2020), where a class of T cells secretes acetylcholine and inhibits the production of inflammatory cytokines by splenic macrophages (Rosas-Ballina et al., 2011). When this anti-inflammatory regulatory response works properly, it limits the spread of viral infection and is essential for controlling and addressing the infection and its inflammatory consequences. However, in the case of low parasympathetic nerve activity, the inflammatory response may go unchecked, leading to excessive inflammation, commonly known as a “cytokine storm” (Fajgenbaum and June, 2020). In addition, during induced infection, parasympathetic nerve stimulation was associated with increased natural killer activity, CD4+ (Newell-Rogers et al., 2022), and CD8+ cells (Carnevale et al., 2020). Therefore, the parasympathetic nerve on the one hand controls inflammation, and on the other hand, can increase antiviral immunity.

Immunoglobulin A is the main component of the body’s mucosal defense system (Isho et al., 2021), widely distributed in milk, saliva, gastrointestinal tract, respiratory tract, and urogenital tract mucosal secretions. It can inhibit the adhesion of microorganisms in the respiratory epithelium, slow down the reproduction of viruses, exhibit an important immune barrier effect, demonstrate antibody activity against certain viruses, bacteria, and general antigens, and serve as the first line of defense against pathogen invasion. From our test results, salivary IgA levels in the music group were significantly higher than those in the control group (Figure 5). Therefore, it is suggested that music may exert immunomodulatory roles by lowering cortisol levels and increasing IgA levels. Thus, the specific mechanism by which music regulates immune function through the autonomic nervous system still needs to be further explored. However, our data suggest a causal relationship between music-induced sympathetic/parasympathetic balance and endocrine-immune homeostasis.

Our new music combination also improves mental function. Volunteers in the music group not only improved their attention and attention span but also became more decisive in the BART results (Figure 4). The measurement mainly focuses on reward and emotion-oriented risk decision-making behaviors, which are regulated by cognitive control and attention-related brain regions. Based on this experiment, we hypothesize that the changes in the BART measurement results of the music group’s volunteers may be mainly related to music’s regulation of emotions, such as stress reduction, and potentially related to the music’s influence on cognition. In Bleibel et al.’s (2023) recent review, out of eight studies on the cognitive effects of music on Alzheimer’s disease (AD) patients, seven of them found that music therapy had a significant positive effect on enhancing cognitive function in AD patients. Although we did not compare the effects of the new music combination used here with other music, our data showed that the music we used did improve the homeostasis of the neuroendocrine and immune system and improve cognition to some extent.

Music therapy has two primary methods: active music therapy, where the music therapist uses improvisational, re-creative, or compositional techniques, and receptive music therapy, where the patient serves as a recipient of the music experience (Aalbers et al., 2017). In receptive music therapy, the music could be selected either by the experimenters or by the volunteers (Bleibel et al., 2023). Previous reports including ours found that Mozart K.448 (Di Cesare et al., 2022; Wang et al., 2025) and Chinese music(Bekiroğlu et al., 2013; Bradt et al., 2013; Chanda and Levitin, 2013; Dallı et al., 2023; Di Cesare et al., 2022; Lee, 2016; Li et al., 2010, 2022; Mol et al., 2021; Pauwels et al., 2014; Tao et al., 2016; Wang et al., 2025; Zhang et al., 2021) can help recover attention after mental fatigue or have shown stress-relieving effects. Here combining them may avoid fatigue induced by repeated single-track playback and achieve the goal of synergistic enhancement. In this study, we innovatively propose a new music combination as an intervention means for music therapy. This music combination is an innovative combination of Chinese and Western music, based on an extensive literature review and research into traditional Chinese Five-Element music (Zhu et al., 2021).

The cardiac autonomic nervous system primarily influences endocrine function through direct neural innervation of endocrine organs and regulation of hormone release. For example, sympathetic hyperactivity can continuously activate the renin-angiotensin system (RAS), leading to elevated cortisol levels; the parasympathetic nervous system (mainly the vagus nerve), on the other hand, reduces excessive hormone secretion by antagonizing sympathetic activity or acting directly on endocrine cells (Jones et al., 2020; Kunikullaya et al., 2025). Endocrine hormones can regulate immune function through mechanisms such as acting on receptors expressed on immune cells. In this study, we found that cortisol can significantly inhibit the expression of immunoglobulin A (IgA), thereby providing direct evidence for the regulation of the autonomic nerve-endocrine-immune axis (Figure 5).

In this study, we did not consider the participants’ interest in music or any regular listening to music, and music was chosen based on previous reports (Li et al., 2010, 2022; Tao et al., 2016) and our work (Wang et al., 2025), which is a limitation of this study. To control for the effects of the menstrual cycle on hormonal and autonomic function, only male subjects were included to reduce intergroup variance, which could create gender bias. This design limits the replicability and generalizability of the study’s conclusions to female populations. The next studies would include more diverse people in future studies.

## 5 Conclusion

Here, we found that the new music combination increased the activity of the parasympathetic nerve as marked by modulating HRV, which led to lower BART scores and higher MOT test scores, suggesting stress relief. In addition, our new music combination promotes endocrine-immune homeostasis, as marked by increased IgA levels and decreased cortisol levels. Our data suggested that music can also work by affecting the peripheral nervous system and supports the use of HRV to evaluate the effects of music on neuroimmune disorders, and suggests the causal relationship between music-induced sympathetic/parasympathetic balance and endocrine-immune homeostasis.

Music has been widely used for disease intervention. This study showed that the novel music combination can relieve stress in high-intensity work during the day, promote endocrine-neuroimmune homeostasis. Music intervention can function as a stress reliever to improve health. It showed a new direction in the mechanisms of music therapy. The function and mechanism of the music still need to be further explored and expanded.

## Data Availability

The raw data supporting the conclusions of this article will be made available by the authors, without undue reservation.

## Supplementary material

Supplementary Table 1 HRV Data.
